# The first Western Palearctic record of *Euprosthenops* Pocock (Araneae, Pisauridae), with description of a new species from Israel

**DOI:** 10.3897/zookeys.1065.74119

**Published:** 2021-10-22

**Authors:** Sergei Zonstein, Yuri M. Marusik

**Affiliations:** 1 Steinhardt Museum of Natural History, Klausner 12, 69978 Tel-Aviv, Israel Steinhardt Museum of Natural History Tel-Aviv Israel; 2 Institute for Biological Problems of the North RAS, Portovaya Str.18, Magadan, Russia Institute for Biological Problems of the North RAS Magadan Russia; 3 Department of Zoology & Entomology, University of the Free State, Bloemfontein 9300, South Africa University of the Free State Bloemfontein South Africa

**Keywords:** Afrotropical, Arava Valley, new species, paleotropical, spiders, taxonomy

## Abstract

The primarily Afrotropical genus *Euprosthenops* Pocock, 1897 is recorded in the Western Palearctic for the first time. A diagnosis and an illustrated description of *E.insperatus***sp. nov.**, based on a single male from southern Israel, are provided. Considering the structure of the male palp, the holotype of *E.insperatus***sp. nov.** resembles males of two widespread African species, *E.australis* Simon, 1898 and *E.proximus* Lessert, 1916; it differs from them by colouration pattern as well as by the different shapes of the retrolateral tibial apophysis and the palpal sclerites. A short survey of the regional insect and spider genera of the paleotropical origin is also presented.

## Introduction

The spider genus *Euprosthenops* Pocock, 1897 currently includes nine species and one subspecies distributed within the mainland Sub-Saharan Africa except one species known from India and Pakistan (WSC 2021). The genus is relatively well studied due to the surveys by Blandin (1974, 1975, 1976, 1978) and Silva and Sierwald (2014). However, little attention has been paid to the disjunct distribution of the genus, with a wide gap between the ranges of Afrotropical and Indo-Malayan species. The present study is based on a quite unexpected occurrence of a single male congener in the Arava Valley, southernmost Israel. After examination, the male has been considered to represent a new species of *Euprosthenops*, which is diagnosed, described and illustrated herein.

## Material and methods

### Acronyms

**NMW**Naturhistorisches Museum Wien, Vienna, Austria;

**SMNH** Steinhardt Museum of Natural History, Tel-Aviv, Israel.

Comparative material: Euprosthenopssp. aff.australis Simon, 1898 – 1♂ (NMW), Namibia, Windhoek (no other data). *Euprosthenopsproximus* Lessert, 1916 – 1♂ (SMNH), DR Congo, *Bandundu Province*: Salonga Nat. Park, Lokoro River basin, about 110 km SSW Monkoto Village, 2°45.8'N, 20°19.3'E, alt. 400 m, 1.01–15.02.2018 (V. Kravchenko & G. Müller).

Photographs were taken using an Olympus SZX16 stereomicroscope with a Canon EOS 7D camera and prepared using the Helicon Focus 7.6.2 Pro (http://www.heliconsoft.com). Measurements were taken through the above-mentioned stereomicroscope to an accuracy of 0.01 mm. All measurements are given in millimetres.

### Abbreviations

**ALE** anterior lateral eye(s);

**AME** anterior median eye(s);

**PLE** posterior lateral eye(s);

**PME** median lateral eye(s).

Other used abbreviations are explained in the text and in the captions.

## Taxonomy

### Family Pisauridae Simon, 1890

#### Genus Euprosthenops

Taxon classificationAnimaliaAraneaePisauridae

Pocock, 1897

805E5151-7A43-5E14-8954-371805936B21

##### Type species.

*Podophthalmabayonianna* Brito Capello, 1867, by subsequent designation (Simon 1898).

##### Diagnostic characters.

The genus and its characters were comprehensibly described by Blandin (1976) and later redescribed by Silva and Sierwald (2014). Among the used characters (for their full set see Silva and Sierwald 2014), two are principal in distinguishing males from those of the closely related genus *Euprosthenopsis* Blandin, 1974. First, in males of *Euprosthenops* the palpal tibia is armed with a flattened and extended chisel-shaped retrolateral tibial apophysis (*Rta*; see Fig. 2C, D). Second, they possess a large lamellose distal tegular apophysis (*Dt*; Figs 2C, 4, 5). On the contrary, males of *Euprosthenopsis* have a wide and concave retrolateral tibial apophysis as well as a short and rounded distal tegular apophysis (see Blandin 1974; Silva and Sierwald 2014).

##### Composition and distribution.

According to WSC (2021) with the present addition, *Euprosthenops* includes ten species and one subspecies: ♂♀ *E.australis* Simon, 1898 (Senegal, Nigeria, Zambia, Botswana and South Africa), ♂♀ *E.bayaonianus* (Brito Capello, 1867) (West, Central and East Africa), ♀ *E.benoiti* Blandin, 1976 (Rwanda), ♂♀ *E.biguttatus* Roewer, 1955 (Congo, Namibia), ♂♀ *E.ellioti* (O. Pickard-Cambridge, 1877) (India, Pakistan?), ♂ *E.insperatus* sp. nov. (Israel), ♀ *E.pavesii* Lessert, 1928 (Central and East Africa), ♂♀ *E.proximus* Lessert, 1916 (Central, East and South Africa), ♂♀ *E.p.maximus* Blandin, 1976 (Ivory Coast), ♀ *E.schenkeli* (Roewer, 1955) (East Africa), ♂ *E.wuehlischi* Roewer, 1955 (Namibia). The record of a single female specimen of *E.ellioti* in the Pakistani Punjab by Dyal (1935) is doubtful, as there are no illustrations provided for this material and it is possible that even the generic assignment is not correct.

#### Euprosthenops insperatus

Taxon classificationAnimaliaAraneaePisauridae

sp. nov.

303769F2-FFDE-55DE-AB14-6AD7B966E2C2

http://zoobank.org/21B8E295-1DE4-4B5D-9907-A413F3203AA4

**Figure 1.**
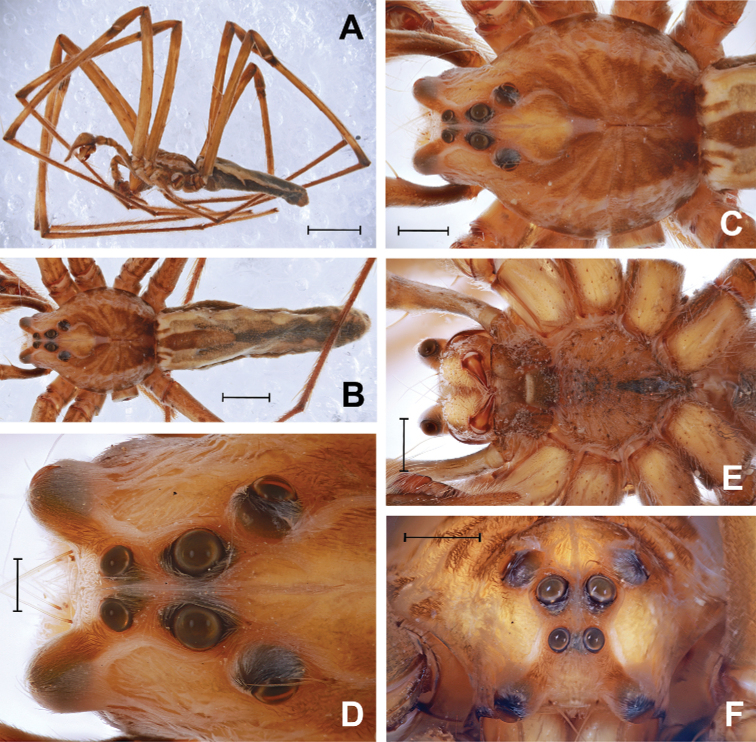
*Euprosthenopsinsperatus* sp. nov., holotype male **A, B** habitus, dorsal and lateral **C, E** cephalothorax, dorsal and ventral **D, F** eye group, dorsal and frontal. Scale bars: 5 mm (**A**); 2 mm (**B**); 1 mm (**C, E, F**); 0.5 mm (**D**).

**Figure 2.**
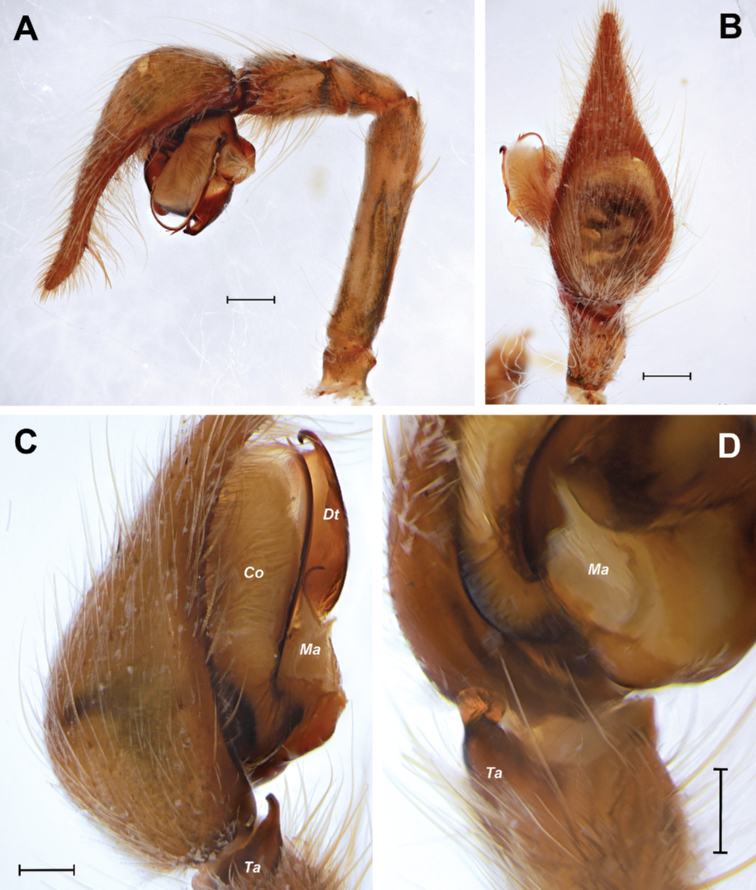
*Euprosthenopsinsperatus* sp. nov., holotype male, structures of left (**A, B**) and right (**C, D**) palp **A** entire palp, retrolateral **B** palpal tibia and cymbium, dorsal **C, D** distal palpal tibia and basal embolus close up, retrolateral and ventral. Abbreviations: *Co* – conductor, *Dt* – distal tegular process, *Ma* –– median apophysis, *Ta* – retrolateral tibial apophysis. Scale bars: 0.5 mm (**A, B**); 0.2 mm (**C, D**).

Figs 1 , 2 , 4A–D , 5A, C , 7 , 8

##### Type material.

***Holotype*** ♂ (SMNH), Israel, Southern District: Arava Valley, Hahal Shezaf 5 km S. Hazeva (Hatseva) Village, 30°43'N, 35°16'E, –120 m (below sea level), 26.03.2006 (S. Zonstein). The spider was collected within the Aqaba–Jordan section of the East African – Syrian rift zone, in a few kilometres to the west from the midline of fault. The holotype specimen is in a relatively good condition, only the left leg III is completely missed being evidently lost prior to sampling and preservation.

##### Diagnosis.

**Figure 3.**
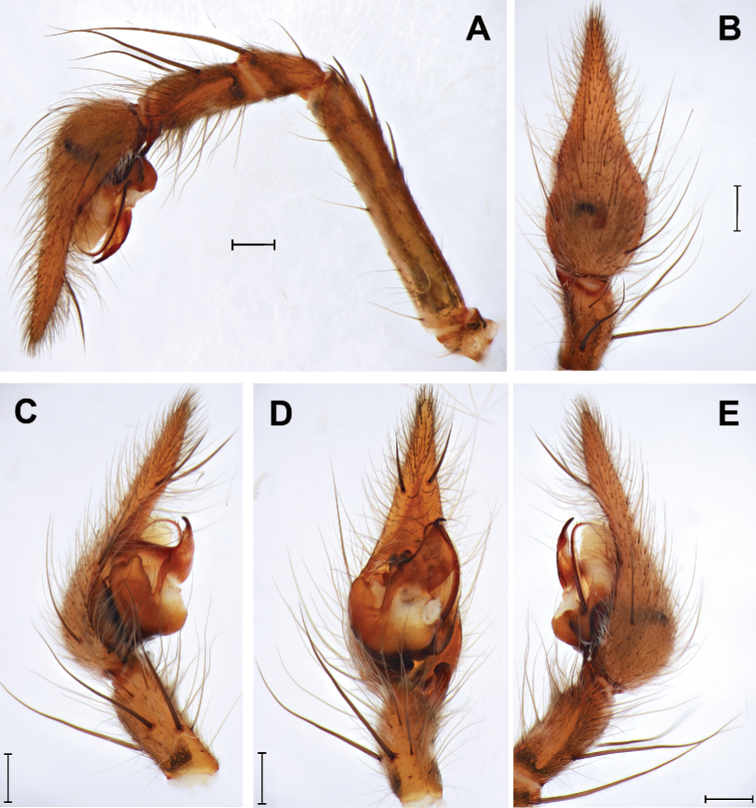
*Euprosthenopsproximus* Lessert, 1916, male, structures of left palp **A** entire palp, retrolateral **B–E** palpal tibia and cymbium, dorsal, prolateral, ventral and retrolateral. Scale bars: 0.5 mm.

**Figure 4.**
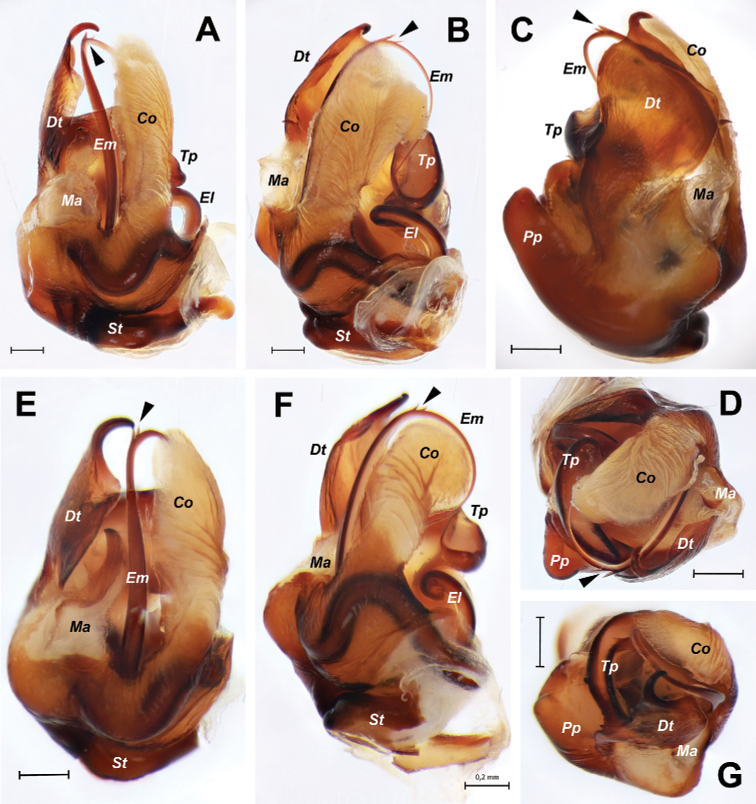
*Euprosthenopsinsperatus* sp. nov., holotype male (**A–D**) and *E.proximus* Lessert, 1916, male (**E–G**), separated copulatory bulb **A, E** retrolateral **B, F** retrodorsal **C** ventral **D, G** frontal. Small teeth of embolus are indicated with arrows. Abbreviations: *Co* – conductor, *Dt* – distal tegular process, *El* – embolus loop, *Em* – embolus, *Ma* – median apophysis, *Pp* – prolateral projection, *St* – subtegulum, *Tp* – prolateral tegular pouch. Scale bars: 0.2 mm.

**Figure 5.**
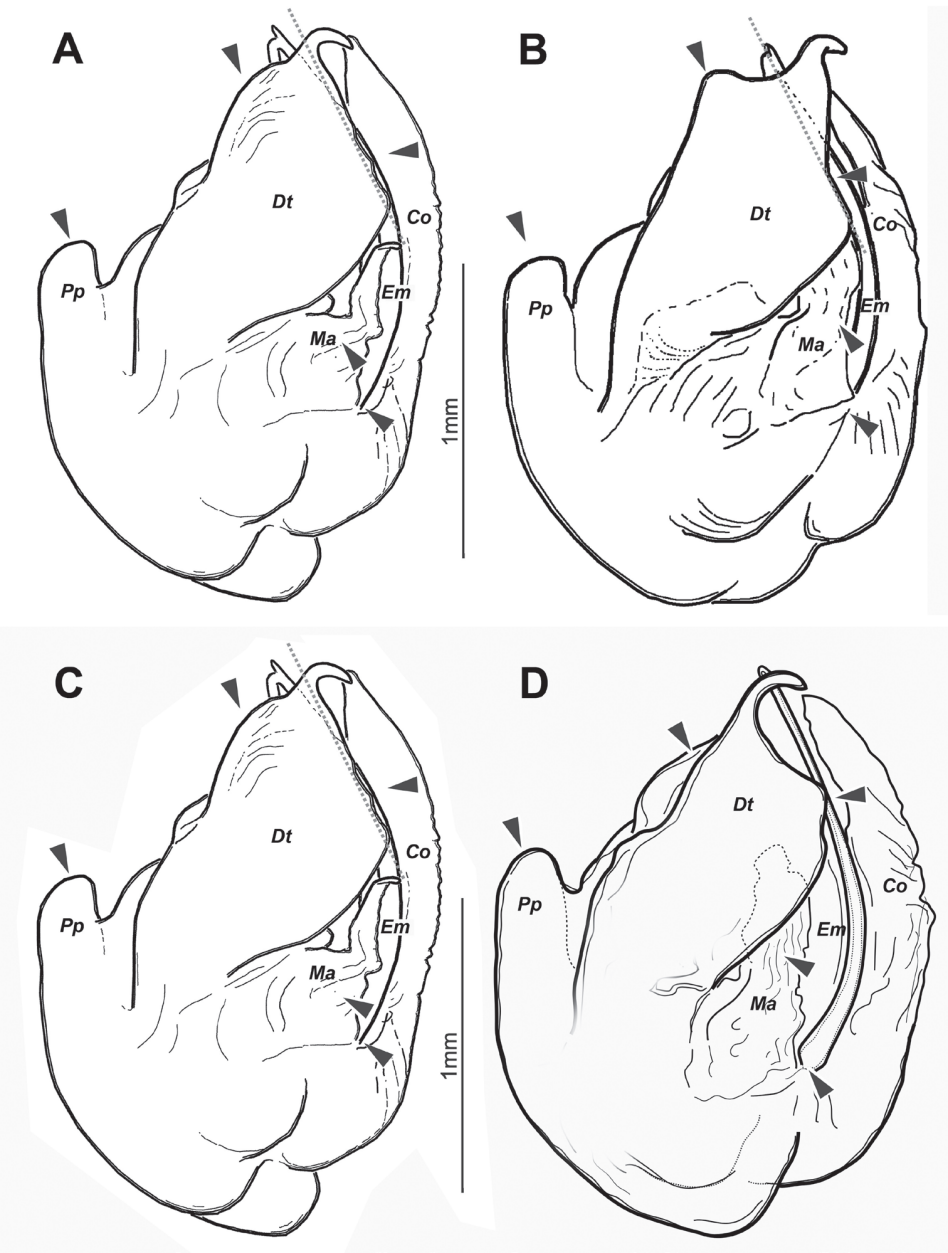
*Euprosthenopsinsperatus* sp. nov., holotype male (**A, C**), and males of *E.australis* Simon, 1898 (**B**) and *E.proximus* Lessert, 1916 (**D**); comparison of copulatory bulbs schematically depicted in the same position. The differences in their structure are indicated with arrows. Abbreviations: *Co* – conductor, *Dt* – distal tegular process, *Em* – embolus, *Ma* –– median apophysis, *Pp* – prolateral projection.

The sole male of the new species most closely resembles the males of *E.australis* and *E.proximus* in a number of similarly shaped structures: the distal tegular apophysis (*Dt*), the tegular prolateral projection (*Pp*), the median apophysis (*Ma*) and the retrolateral tibial apophysis (*Ta*). *Euprosthenopsinsperatus* sp. nov. differs from these similar species in having relatively longer prolateral tegular projection (length of tegulum/length of projection ratio 1.4 to 1.5 vs. 1.6), in the localization of the embolus origin (posteriorly from posterior edge of distal tegular apophysis vs. anteriorly in *E.australis*), and in the shape of the distal part of the distal tegular apophysis, as well as by shorter palpal tibia (length/width ratio 1.5 vs. 1.6 to 1.7). Structure of male palp differs in many details from that in *E.proximus* and *E.australis* (Figs 2, 4A–F, 5A, C cf. Figs 3, 4E–G, 5B, D). From *E.schenkeli*, *E.pavesii* and *E.benoiti*, where the conspecific males remain unknown, *E.insperatus* sp. nov. can be distinguished by having a dissimilar dorsal abdominal pattern (Fig. 1B cf. Blandin 1976, figs 2, 3, 8).

##### Description.

**Male.** Habitus as in Fig. 1A, B. Total body length 13.75. Color in alcohol: cephalothorax, chelicerae, palps and legs mostly light to medium ginger brown; X-shaped eye group and radial thoracic grooves darkened; eyes encircled with narrow blackish areas; postocular area, chelicerae anteriorly and coxae I–IV ventrally light yellowish brown; maxillae and labium medium brown, sternum medium brown with short longitudinal dark brown band posteriorly; abdomen light brown anterodorsally, other parts of abdomen dark brown; carapace with two wide submarginal bands of adpressed whitish pubescence, abdomen with two similar longitudinal bands dorsally and with two very narrow light grey bands ventrally; all segments of palps and legs I–IV slightly to noticeably darkened proximally and subapically.

Carapace (Fig. 1C) 5.45 long, 4.21 wide. Clypeus and eye group as in Fig. 1D, F. Clypeus height 0.62. Eye diameters and interdistances: ALE 0.33, AME 0.22, PME 0.36, PLE 0.35, ALE–ALE 1.21, ALE–AME 0.75, AME–AME 0.16, AME–PME 0.33, ALE–PLE 1.64, PME–PME 0.23, PME–PLE 0.45, PLE–PLE 1.27. Cheliceral fang furrow: promargin and reromargin each armed with narrow row of 3 evenly disposed teeth, promargin with smaller uniformly sized and shaped teeth; within unevenly larger teeth of retromarginal row, median tooth largest. Sternum, labium and maxillae as in Fig. 1E. Labium 0.69 long, 0.86 wide. Sternum sharply nonagonal, 2.42 long, 2.43 wide.

Ventral pairs of spines on tibiae I–IV: 4, 4, 3, 4, respectively. Paired claws on tarsi I–IV with 6–7 teeth each.

Palp and leg measurements as follows:

**Table:** 

	**Femur**	**Patella**	**Tibia**	**Metatarsus**	**Tarsus**	**Total**
Palp	2.74	0.85	0.96	—	2.91	7.46
Leg I	11.31	2.90	11.84	12.77	5.66	44.48
Leg II	10.74	2.89	10.53	11.84	5.29	41.29
Leg III	9.13	2.18	7.98	8.27	3.71	31.27
Leg IV	11.28	2.54	11.01	11.15	4.89	40.87

Male palp (Figs 2, 4A–D, 5A). Femur shorter than cymbium, 5 times longer than wide. Tibia slightly longer than patella with retrolateral apophysis (*Ta*) shorter than tibia’s width. Cymbium 2.35 longer than wide, with long tip (about 0.25 of cymbium length). Subtegulum (*St*) moderately small and located retrolaterally. Tegulum with prolateral pouch (*Tp*) and distinct prolateral projection (*Pp*), height of projection (from base of tegulum to tip of projection) exceeds length of tegular distal apophysis (*Dt*). Distal apophysis with prolateral hook-shaped tip, anterior prolateral part slanting. Conductor (*Co*) large and long, weakly sclerotized. Embolus with 2 teeth on anterior loop.

**Female.** Unknown.

##### Etymology.

From the Latin adjective of the masculine gender “*insperatus*” for “unforeseen”, alluding to the unexpected discovery of a species belonging to the previously paleotropical genus *Euprosthenops* in Israel.

##### Ecology.

**Figure 6.**
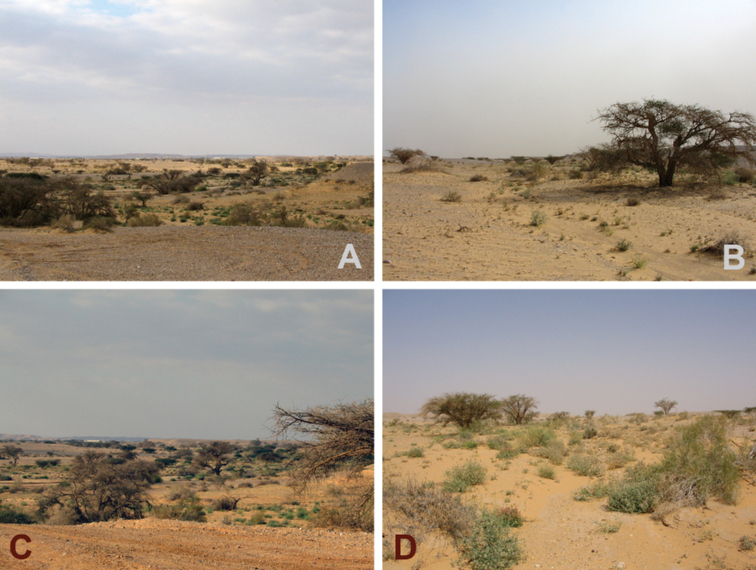
Surroundings of Nahal Shezaf, the type locality of *Euprosthenopsinsperatus* sp. nov. (**A–D**).

The holotype was found inside a small patch of shrubs and reeds growing close to a periodically wet riverbed that crosses the extremely arid desert biotopes (Fig. 6).

##### Distribution.

**Figure 7.**
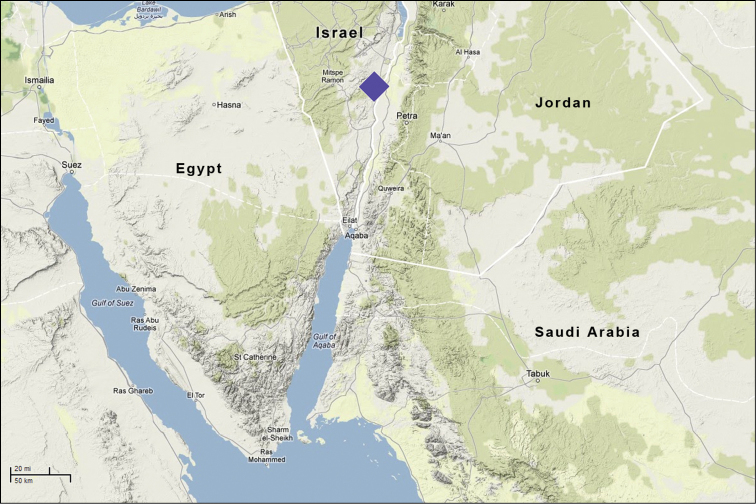
*Euprosthenopsinsperatus* sp. nov., distribution record.

**Figure 8.**
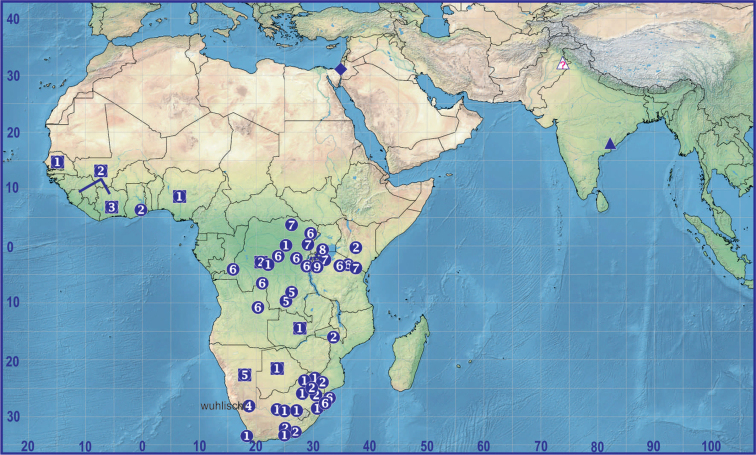
Distribution of *Euprosthenops* spp. Records of Afrotropical congeners are presented as circles (localities) and quadrangles (country records) according to numbers: **1***E.australis* Simon, 1898 **2***E.bayaonianus* (Brito Capello, 1867) **3***E.proximusmaximus* Blandin, 1976 **4***E.wuehlischi* Roewer, 1955 **5***E.biguttatus* Roewer, 1955 **6***E.proximusproximus* Lessert, 1916 **7***E.pavesii* Lessert, 1928 **8***E.schenkeli* (Roewer, 1955) **9***E.benoiti* Blandin, 1976. Records of Asian *E.ellioti* (O. Pickard-Cambridge, 1877) and *E.insperatus* sp. nov. are indicated as triangles (? means questionable record) and diamond, respectively.

Known only from the type locality (Fig. 7). The location of this sole record in relation to the records of other congeners lays far outside the previously known genus range (Fig. 8).

## Discussion

Since the 1960s, the territory belonging to the modern Israel is known as the “crossroads” for different plant and animal taxa penetrating the country from the north, south and east (Zohary 1966; Danin et al. 1975; Furth 1975; Danin 1988; Freidberg 1988; Tchernov 1988). This biogeographical feature of the country can explain why species of paleotropical origin, primarily associated with the adjacent regions of East Africa, are presented here. The data listed below do not claim to be exhaustive; they are merely intended to show that a pattern of distribution, similar to the above-noted one, is neither frequent nor exclusive in relation to various groups of spiders and insects represented in Israel and adjacent Middle East countries.

Regarding the spiders (Araneae), the genus *Levymanus* Zonstein & Marusik, 2013 (Palpimanidae) originally was established as a monotypic taxon known only from the desert rift zone in south Israel (see Zonstein and Marusik 2013). Later, its type species was found in Saudi Arabia (El-Hennawy 2014) and UAE (Zonstein et al. 2017). Finally, two additional species were described from Ethiopia and south Iran (Zonstein et al. 2017; Zamani and Marusik 2020).

Two widespread paleotropical genera, *Calommata* Lucas, 1837 (Atypidae) and *Cambalida* Simon, 1909 (Corinnidae) are represented in Israel by a single species each (Levy 2007; personal unpublished data regarding the presence of *Cambalida* sp. in Israel). The shared range of all other species belonging to these genera extends from the Sub-Saharan Africa to south and eastern Asia (WSC 2021). Likewise, *Evipommasimoni* Alderweireldt, 1992 is a representative of the mostly paleotropical wolf spider genus *Evipomma* Roewer, 1959 (Lycosidae); this species, known previously from Sudan and Egypt, has been very recently found in southern regions of Israel (see Armiach Steinpress et al. 2021).

The similar situation is observed in two Afrotropical spider genera. Within eight species of *Festucula* Simon, 1901 (Salticidae) known from Sub-Saharan Africa, the generotype *E.vermiformis* Simon, 1901 has been recorded also in Sudan, Egypt and Israel (Azarkina and Foord 2014; WSC 2021). The same is true for *Pararaneus* Caporiacco, 1940 (Araneidae), where four of five species are limited in their distribution to either the mainland Africa or Madagascar, and only the trans-African *P.spectator* (Karsch, 1885) extends northward the genus range to Yemen, Sinai (Egypt) and Israel (Levy 1998; WSC 2021).

A similar disjunct distribution is recorded for the mostly Afrotropical huntsman spider genus *Pseudomicrommata* Järvi, 1912 (Sparassidae). Here, the distribution of four congeners is restricted to the western, eastern and southern regions of the mainland Africa (Moramand 2015). However, one species, *P.mocranica* Moramand, Zamani & Jäger, 2019 has been recently found in the Sistan & Baluchistan Province of Iran (see Moramand et al. 2019).

Among the insects (Hexapoda), the distribution of a paleotropical (predominantly, of an Afrotropical) taxon having its northernmost limit in Israel or very close to it can be observed in several insect orders. According to Blondi et al. (2017), the Afrotropical flea beetle genus *Calotheca* Heyden, 1887 (Coleoptera, Chrysomelidae) embraces 27 species distributed predominantly in central, southern and eastern regions of mainland Africa. One of them, *C.sacra* (Weise, 1897), is known mostly from East Africa (Eritrea, Ethiopia, Sudan), with one record in southwestern Saudi Arabia. However, this species considerably extends the genus range, penetrating the Great Rift Valley to the north as far as the northern coast of the Dead Sea (where it was originally described from).

In the tiger beetle subfamily Cicindelinae (Carabidae), East African *Cephalotalittorea* (Forskål, 1775) spreads northward almost achieving the Egypt-Israeli border at the northwestern coast of the Gulf of Aqaba, while Asian records of *Habroderanylotica* (Dejean, 1825) distributed throughout the mainland Africa and recorded also for the Canary Islands, are limited to the Sinai mountains (Matalin and Chikatunov 2016, fig. 5).

Within the weevil family Curculionidae, the paleotropical (and mostly Afrotropical) genus *Aorus* Schoenherr, 1835 is represented in Israel by *A.anthracinus* Brancsik, 1898, and this sole Palearctic record is the northernmost point of the genus distribution (Friedman 2018). A similar situation is observed in the African weevil genus *Bradybibastes* Heller, 1923, where one of species, *B.discoidalis* (Tournier, 1873), was found also in the southern part of Israel (see Friedman 2009). Inside the species-rich weevil genus *Merus* Gistel, 1857, *M.friedbergi* Friedman, 2019, recently described from south Israel, is the only Palearctic member of the *denticulatus* species group. So far, this species group has been considered to include 10 described and a few undescribed species from east, west, central and south regions of Africa, with the majority of the species concentrating along the Great Rift Valley (Friedman 2019). According to Friedman (2009), two apparently undescribed weevil species belonging to the Afrotropical genus *Cylindroides* Fairmaire, 1886 occur in southern Israel in the Rift Valley and in the Central and Southern Negev.

In the mayfly family Baetidae (Ephemeroptera), *Cloeonperkinsi* Barnard, 1932, previously known only from the western, eastern and southern regions of the mainland Africa, has been very recently found in Yemen, western Saudi Arabia and Israel (Yanai et al. 2020).

Among the taxa of Diptera, a disjunctive fruit fly genus *Hyalotephritis* Freidberg, 1979 includes only two species: *H.planiscutellata* (Becker, 1903), originally described from Egypt and then found in Ethiopia and Israel, and *H.complanata* (Munro, 1929) known from South and South-Western Africa (Freidberg 1979). The robber fly genus *Lamyra* Loew, 1851 (Asilidae), which has been recently revised and relimited to four species, is endemic to the Afrotropical Realm; however, one of these species, *L.vorax* Loew, 1858, extends into Israel, Yemen, UAE and Saudi Arabia in the Palearctic Region (Dikow and Londt 2000).

Similar paleotropical relations are also known for some Israeli taxa of moths and butterflies (Lepidoptera). Since description, the tiger moth genus *Olepa* Watson, 1980 (Erebidae, Arctiinae) has been considered as restricted to South and South-Eastern Asia (see Singh and Singh 2013). Several years ago, however, the first Palearctic species certainly belonging to the genus was described from Israel (Witt et al. 2005). Between 420 species of the butterfly family Noctuidae, registered in Israel by 2007, only two species of *Condica* Walker, 1856 follow this type of distribution (see Kravchenko et al. 2007). According to these data, *C.capensis* (Gueneé, 1852), widespread in the Old World tropical zone, penetrates Arabian Peninsula, Egypt and Israel, with the northernmost limit of its range in the Dead Sea area. While the mostly South Asian *C.palestinensis* (Staudinger, 1895) spreads northward along the rift zone to the Jordan Valley and Syria.

All the above-noted examples indicate that the disjunctive range of *Euprosthenops* is only a particular case of a more common pattern. In the future, either *E.insperatus* sp. nov. itself or related species, could well be found in Egypt, Yemen, Saudi Arabia and other regions of the Middle East. It is possible, however, that for various reasons, the former connections have disappeared and the gap will remain unfilled.

## Supplementary Material

XML Treatment for Euprosthenops

XML Treatment for Euprosthenops insperatus

## References

[B1] Armiach Steinpress I Alderweireldt M Cohen M Chipman A Gavish-Regev E (2021) Synopsis of the Evippinae (Araneae, Lycosidae) of Israel, with description of a new species. European Journal of Taxonomy 733: 87‒124. 10.5852/ejt.2021.733.1225

[B2] Azarkina GN Foord SH (2014) A revision of the Afrotropical species of *Festucula* Simon, 1901 (Araneae: Salticidae). African Invertebrates 55(2): 351‒375. 10.5733/afin.055.0201

[B3] Blandin P (1974) Etudes sur les Pisauridae africaines II. Définition du genre *Euprosthenops* Pocock, 1897 et description du genre *Euprosthenopsis* n. gen. (Araneae – Pisauridae – Pisaurinae). Revue de Zoologie Africaine 81: 933‒947. 10.5962/bhl.part.76052

[B4] Blandin P (1975) Note sur *Euprosthenopsellioti* (Pickard-Cambridge O., 1877) (Araneae – Pisauridae – Pisaurinae). Bulletin de la Société Zoologique de France 100(4): 575–581.

[B5] Blandin P (1976) Etudes sur les Pisauridae africaines IV. Les espèces du genre *Euprosthenops* Pocock, 1897 (Araneae – Pisauridae – Pisaurinae). Revue Zoologique Africaine 90(1): 63–88.

[B6] Blandin P (1978) Le problème de l’espèce chez les araignées. Les problèmes de l’espèce dans le règne animal 2: 13–56.

[B7] Blondi M Frasca R Grobbelaar E D’Alessandro P (2017) Supraspecific taxonomy of the flea beetle genus *Blepharida* Chevrolat, 1836 (Coleoptera: Chrysomelidae) in the Afrotropical Region and description of Afroblepharida subgen. nov. Insect Systematics & Evolution 48: 97–155. 10.1163/1876312X-48022152

[B8] Danin A (1988) Flora and vegetation of Israel and adjacent areas. In: Yom-Tov Y Tchernov E (Eds) The zoogeography of Israel.Dr. W. Junk Publishers, Dordrecht, 129–158.

[B9] Danin A Orshan G Zohary M (1975) The vegetation of the northern Negev and the Judean Desert of Israel. Israel Journal of Botany 24: 118–172.

[B10] Dikow T Londt JGH (2000) A review of *Lamyra* Loew (Diptera: Asilidae: Laphriinae). African Entomology 8(2): 189–200.

[B11] Dyal S (1935) Fauna of Lahore. 4.–Spiders of Lahore. Bulletin of the Department of Zoology of the Panjab University 1: 119–252.

[B12] El-Hennawy HK (2014) The first record of *Levymanusgershomi* in Saudi Arabia (Araneae, Palpimanidae). Serket 14(2): 97–101.

[B13] Freidberg A (1979) The Afrotropical species assigned to *Terellia* R. D. (Diptera: Tephritidae). Journal of the Washington Academy of Sciences 69(4): 164–174.

[B14] Freidberg A (1988) Zoogeography of Diptera in Israel. In: Yom-Tov Y Tchernov E (Eds) The zoogeography of Israel.Dr. W. Junk Publishers, Dordrecht, 251–276.

[B15] Friedman A-L-L (2009) Review of the biodiversity and zoogeographical patterns of the weevils (Coleoptera, Curculionoidea) in Israel. In: Neubert E Amr Z Taiti S Gümüs B (Eds) Animal Biodiversity in the Middle East. Proceedings of the First Middle Eastern Biodiversity Congress, Aqaba, Jordan, 20–23 October 2008.ZooKeys 31: 133–148. 10.3897/zookeys.31.123

[B16] Friedman A-L-L (2018) Review of the hygrophilous weevils in Israel (Coleoptera: Curculionoidea). Diversity 2018, 10(3): 1–48. 10.3390/d10030077

[B17] Friedman A-L-L (2019) The first record of the genus *Merus* Gistel (Curculionidae: Molytinae: Mecysolobini) in the Western Palaearctic, with description of *Merus freidbergi* n. sp. from Israel. Israel Journal of Entomology 49(2): 351–364. 10.5281/zenodo.3593312

[B18] Furth DG (1975) Israel, a great biogeographic crossroads. Discovery 11(1): 2–13.

[B19] Kravchenko VD Fibiger M Hausmann A Müller GC (2007) The Lepidoptera of Israel. Volume 2. Noctuidae. Pensoft Publishers, Sofia–Moscow, 320 pp.

[B20] Levy G (1998) Twelve genera of orb-weaver spiders (Araneae, Araneidae) from Israel. Israel Journal of Zoology 43: 311–365.

[B21] Levy G (2007) *Calommata* (Atypidae) and new spider species (Araneae) from Israel. Zootaxa 1551: 1–30. 10.11646/zootaxa.1551.1.1

[B22] Matalin AV Chikatunov VI (2016) The tiger beetles (Coleoptera, Carabidae, Cicindelinae) of Israel and adjacent lands. ZooKeys 578: 115–160. 10.3897/zookeys.578.7383 PMC4829963 27110198

[B23] Moradmand M (2015) Revision of the grass huntsman spider genus *Pseudomicrommata* Järvi, 1914 (Araneae: Sparassidae) in the Afrotropical Region. African Invertebrates 56(2): 425–443. 10.5733/afin.056.0213

[B24] Moradmand M Zamani A Jäger P (2019) An Afrotropic element at the north-western periphery of the Oriental Region: *Pseudomicrommatamokranica* sp. nov. (Araneae: Sparassidae). Revue Suisse de Zoologie 126(2): 249–156. 10.5281/zenodo.3463459

[B25] Silva ELC da Sierwald P (2014) Description of the males of *Euprosthenopsaustralis* Simon, 1898 and *Euprosthenopsispulchella* (Pocock, 1902) (Araneae: Pisauridae). Zootaxa 3857(1): 137–150. 10.11646/zootaxa.3857.1.8 25283102

[B26] Simon E (1898) Histoire naturelle des araignées. Deuxième édition, tome second. Roret, Paris, 193–380. 10.5962/bhl.title.51973

[B27] Singh N Singh J (2013) Review of the genus *Olepa* Watson (Lepidoptera: Erebidae: Arctiinae). Tinea 22(4): 272–277.

[B28] Tchernov E (1988) The paleobiogeographical history of the southern Levant. In: Yom-Tov Y Tchernov E (Eds) The zoogeography of Israel.Dr. W. Junk Publishers, Dordrecht, 159–250.

[B29] Witt TJ Müller GC Kravchenko VD Miller MA Hausmann A Speidel W (2005) A new *Olepa* species from Israel (Lepidoptera: Arctiidae). Nachrichtenblatt der Bayerischen Entomologen 54(3/4): 101–115.

[B30] WSC (2021) World Spidwer Catalog. Natural History Museum Bern. http://wsc.nmbe.ch [version 22.0, accessed on 5.05.2021]

[B31] Yanai Z Graf W Terefe Y Sartori M Gattoliat J-L (2020) Re-description and range extension of the Afrotropical mayfly *Cloeonperkinsi* (Ephemeroptera, Baetidae). European Journal of Taxonomy 617: 1–23. 10.5852/ejt.2020.617

[B32] Zamani A Marusik YM (2020) New species of Filistatidae, Palpimanidae and Scytodidae (Arachnida: Araneae) from southern Iran. Acta Arachnologica 69(2): 121–126. 10.2476/asjaa.69.121

[B33] Zohary M (1966) Flora Palaestina. Part I. The Israel Academy of Sciences and Humanities, Jerusalem, 364 pp.

[B34] Zonstein S Marusik YM (2013) On *Levymanus*, a remarkable new spider genus from Israel, with notes on the Chediminae (Araneae, Palpimanidae). ZooKeys 326: 27–45. 10.3897/zookeys.326.5344 PMC3764535 24039534

[B35] Zonstein SL Marusik YM Kovblyuk MM (2017) New data on the spider genus *Levymanus* (Araneae: Palpimanidae). Oriental Insects 51(3): 221–226. 10.1080/00305316.2016.1275989

